# Extensor Medii Proprius: A Cadaveric Case Report

**DOI:** 10.7759/cureus.46018

**Published:** 2023-09-26

**Authors:** Chris Baker, Gabrielle Swartz, Jeanine Garcia, Felix Hernandez Perez, Leslie Pryor McIntosh

**Affiliations:** 1 Biomedical Sciences, Philadelphia College of Osteopathic Medicine, Moultrie, USA; 2 Ecology, Evolution, and Organismal Biology, Kennesaw State University, Kennesaw, USA

**Keywords:** embryology, forearm, anatomic variation, anatomy, clinical, extensor tendons, anomalous extensor, extensor medii proprius

## Abstract

During routine dissection of 11 cadavers that originated with the Body Donor Program at Philadelphia College of Osteopathic Medicine (PCOM) Georgia, a 69-year-old African American male with bilateral extensor anomalies in the dorsal forearm compartment was encountered. The distinct muscle belly, identified as the extensor medii proprius (EMP), originated from the distal ulna and was inserted near the dorsal aponeurosis of the third digit. Manual traction of the right EMP tendon resulted in the extension of the third digit, suggesting the functional significance of the anomalous muscle. This case study analyzes the EMP found during dissection, as well as the anomalous muscle’s prevalence, embryologic origin, and clinical relevance. The presence of the EMP muscle and tendon can be considered when assessing pain in the dorsum of the hand and when preparing for surgical repair or tendon transfer.

## Introduction

Forearm extensor anomalies are relatively common [1]. Identification, recognition, and description of such anomalies are important to add to the existing literature, as they can provide information to medical practitioners on the anatomical variation in the hands for various types of hand surgeries [2]. Additionally, information on the anatomical variation in the human hand is important for researchers looking to describe the functional significance and evolution of the human hand [3]. 

Types of variations previously described in the literature include the extensor digitorum brevis manus (EDBM) [4], the extensor medii proprius (EMP), the extensor indicis et medii communis (EIMC), the extensor pollicis et indicis accessorius (EPI) [2], and variations in the number of extensor digitorum communis (EDC) tendons, slips and their insertion sites [5]. The EDBM is defined as originating from the distal end of the radius and radiocarpal ligaments and inserting into the metacarpophalangeal joint of the third digit [4]. The EIMC is defined as originating from the distal third of the ulna with two tendons inserting into the index and long fingers [6]. The EPI is defined as originating from the distal third of the posterior surface of the ulna and interosseus membrane with two tendons, one inserting into the medial side of the thumb or fused with the extensor pollicis longus proprius tendon and the other inserting into the lateral side of the index finger [7]. 

The normal anatomy of the dorsal forearm consists of six extensor compartments. From radial to ulnar side the order of compartments and their contents is as follows: first compartment contains the abductor pollicis longus and extensor pollicis brevis; second compartment contains the extensor carpi radialis longus and brevis; third compartment contains the extensor pollicis longus; fourth compartment contains the EDC and extensor indicis proprius (EIP); fifth compartment contains the extensor digiti minimi; and sixth compartment contains the extensor carpi ulnaris [8]. The fourth dorsal compartment of the forearm, which typically contains the EIP and EDC tendons, can be the site of supernumerary muscles including the EMP [4,8].

The EMP muscle is an anomalous muscle analogous to the EIP given its adjacent origin but differing in the insertion site [1]. It is identified as having a muscle belly separable from the EIP and insertion into the third digit [1,9]. Anomalous tendons that serve a redundant function can be considered for use in autograft procedures. The EMP can be used in tendon reconstruction to restore abduction of the thumb or extension in the fingers. The second and fifth digits are more likely sources of extra slips/tendons for tendon transfer in surgery as they are more frequently present compared to the EMP [2]. A meta-analytic review of 22 studies with a combined sample size of 3,984 extremities reported the true prevalence of the EMP tendon as 3.7% with higher rates in Japanese and North American populations compared to European and Indian populations [10]. The presence of the EMP rarely causes clinical symptoms due to its narrow width [9].

Here we describe the presence of a bilateral EMP which was discovered during a routine cadaveric dissection. A physical description of the muscle is provided along with a consideration of its prevalence in the cadaveric population studied in the Anatomy Lab at Philadelphia College of Osteopathic Medicine (PCOM) South Georgia. In order to understand the presence and function of this anomalous muscle, its phylogeny and embryological origin are also explored. This research was previously presented as a poster presentation at the Philadelphia College of Osteopathic Medicine Research Week on May 10th, 2021. 

## Case presentation

During the routine dissection of a 69-year-old African American male cadaver, the anomalous EMP muscle was identified in the dorsal forearm compartment of the bilateral upper extremities.

**Figure 1:**
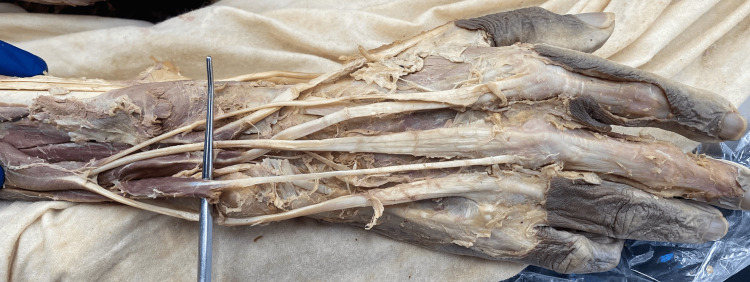
Cadaveric Image of the right upper extremity; the extensor medii proprius (EMP) is overlying the probe

**Figure 2:**
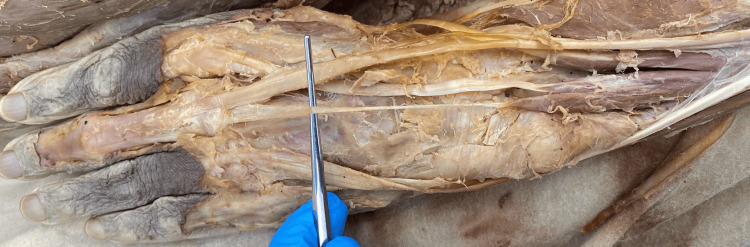
Cadaveric image of the left upper extremity; extensor medii proprius (EMP) is overlying the probe

The origin of the EMP remained constant in bilateral extremities. The origin was distal to the EIP on the distal ulna and interosseous membrane. The insertion site of the EMP observed in both extremities varied. The EMP of the right upper extremity (Figure 1) was inserted into the extensor aponeurosis of the third digit, ulnar to the EDC tendon, and induced extension of the third digit at the metacarpophalangeal (MCP) joint upon manual traction of the tendon. The EMP of the left upper extremity (Figure 2) was inserted into the deep fascia proximal to the MCP joint, ulnar and palmar to the EDC tendon. Manual traction of the left EMP tendon did not produce an extension of the third digit.

Measurements of the EMP in the left and right upper extremities were taken, including tendon width, tendon length, muscle body length, and muscle body width. The tendon length was measured from the insertion site to the myotendinous junction. The muscle body length was measured from the myotendinous junction to the distal-most point of origin. The muscle body width was measured at the widest point in the approximated coronal plane. Measurements were taken with a standard ruler.

The tendon width of the EMP in the right extremity was uniform along its entire length. The tendon width of the EMP in the left extremity increased distally near its insertion site. The proximal tendon of the left EMP muscle was markedly thinner compared to its contralateral counterpart (Table 1).

**Table 1:** Summary of extensor medii proprius (EMP) dimensions

Measurement	Left Upper Extremity	Right Upper Extremity
Tendon Width (mm)	0.11 (proximally) to 0.80 (distally)	0.30
Tendon Length (mm)	10.20	10.40
Muscle Body Length (mm)	6.00	4.10
Muscle Body Width (mm)	0.80	1.70

A total of 21 upper extremities from 11 different cadavers were examined for the presence of the EMP. The EMP was present in two upper extremities of a single cadaver. 

## Discussion

Variations of EMP insertion sites have been reported in the literature, including insertion into the dorsal aponeurosis of the third finger located palmar and ulnar to the insertion of the EDC tendon of the third finger [1] (most common and observed in the current study); insertion into the deep fascia proximal to the MCP located radial to the EDC tendon [1]; and insertion into the intertendinous fascia proximal to the MCP and directly palmar to the EDC tendon [11].

The variability of the insertion site suggests variability in the function of the EMP [1,11]. Depending on its insertion, the EMP can contribute to the extension of the third digit at the MCP joint or extension of the third metacarpal at the wrist. The variance of insertion sites between extremities of a single individual, as observed in this study, appears previously unreported in literature.

The extensor muscles of the forearm differentiate into three distinct layers during early embryologic growth including the radial, superficial and deep layers [12]. Comparative studies suggest that the radial and superficial layers exhibit marked stability while the deep portion appears to be highly unstable and undergoes considerable evolutionary change, as can be seen in the great variation in its expression in different species of primates [12]. The common origin of the EMP and EIP suggests a common embryologic origin of these muscles, being the deep layer of the extensor muscles [1]. The proximal migration of the distal muscle group, consisting of the deep extensor muscles (EIP, EMP), and the distal migration of the proximal muscle group, consisting of the superficial extensor muscles, resulted in an overlap of the function of the two muscle groups [12]. It has been suggested that the EMP became redundant and therefore was lost [1,12]. Therefore, the EMP has been described as an evolutionary remnant as opposed to a variation of a normal arrangement [1]. However, Cauldwell wrote that “it is too easy to point to these variant forms as atavistic or reversions to a common primitive type, when all that can be said with certainty is that they are illustrations and results of a similarity of forces which operate in the evolution of each member of the animal group” [8].

## Conclusions

The EMP muscle was observed bilaterally in the dorsal forearm compartment of a 69-year-old African American male cadaver. Through analysis of the embryologic origins of the EMP, we can further understand its redundant function. Lastly, descriptions of anatomical variation in the forearm and the EMP tendon and can inform alternative surgical approaches to potential sources of tendon grafts.
